# Supplementary figures and images for: Inoculation With a Microbe Isolated From the Negev Desert Enhances Corn Growth
Source: Front Microbiol. 2020 Jun 19;11:1149. doi: 10.3389/fmicb.2020.01149 (PMC7316896; doi:10.3389/fmicb.2020.01149)

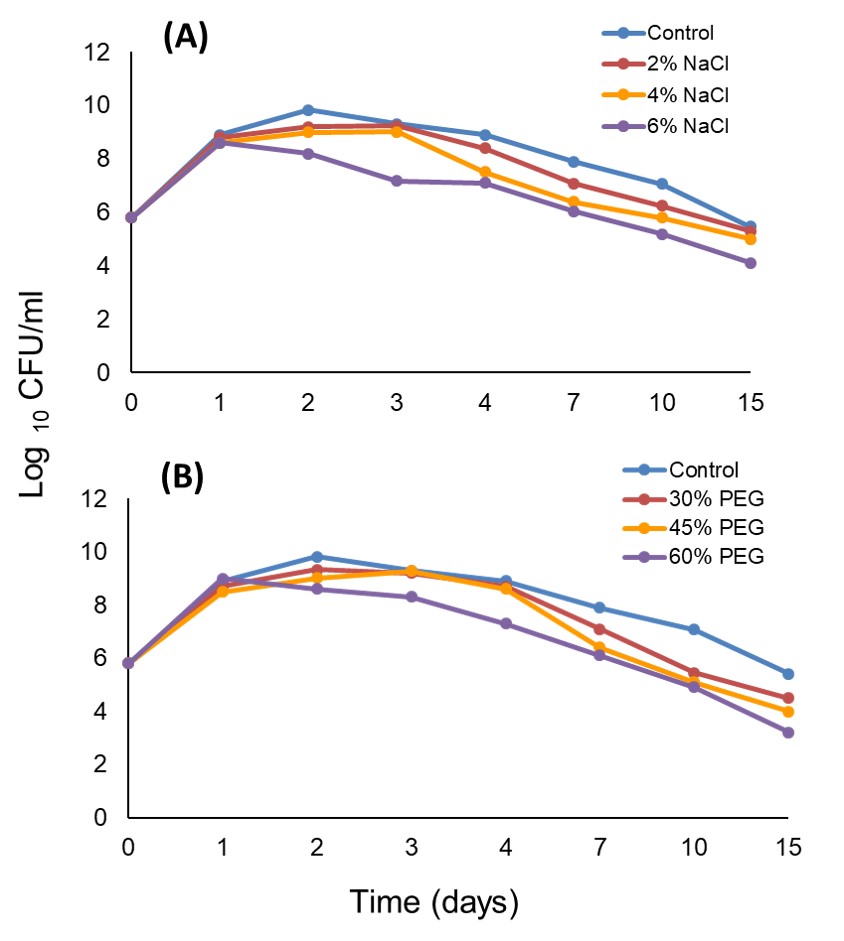

Supplement: FIGURE S1 — Salt (A) and PEG-induced drought (B) stress tolerance of D. cinnamea 55. [file Image_1.JPEG]

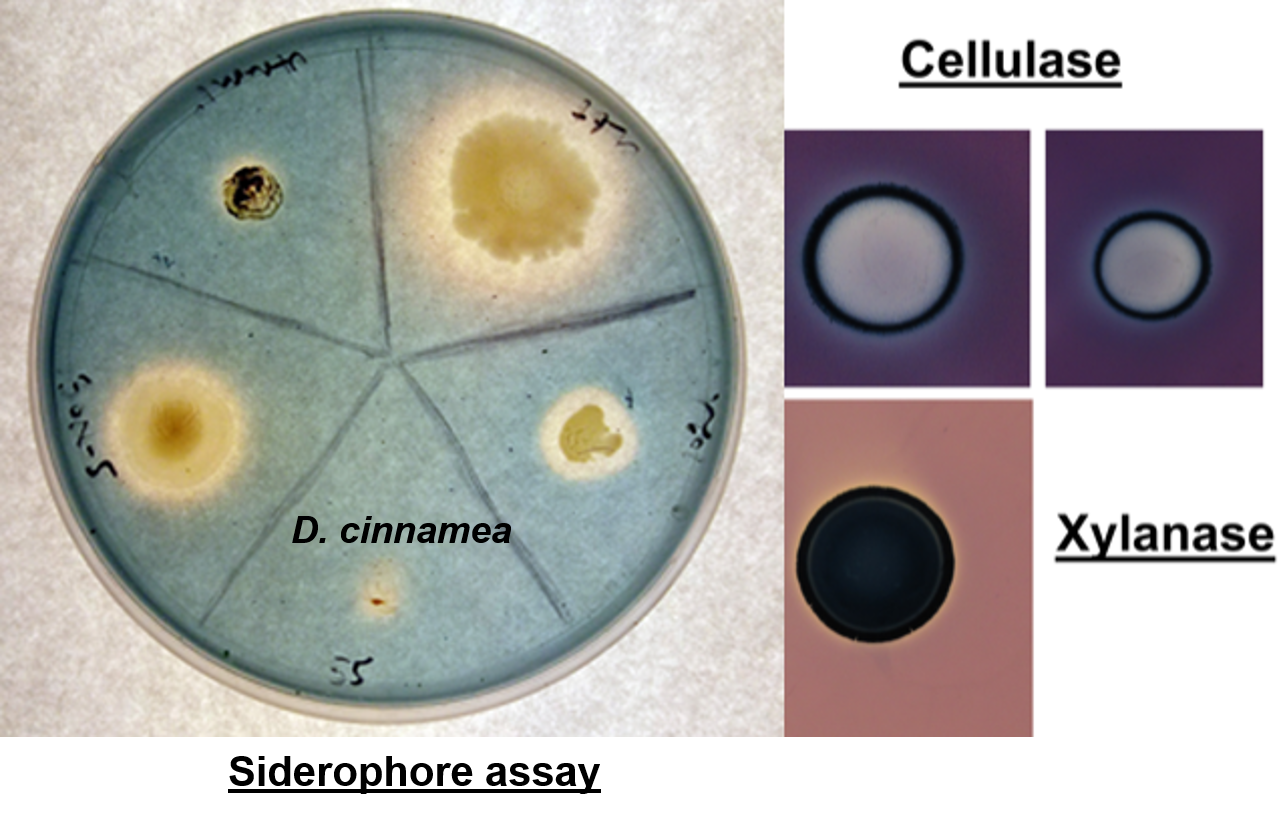

Supplement: FIGURE S2 — Photographs of siderophore production, and xylanase and cellulase activity of D. cinnamea 55. [file Image_2.TIF]

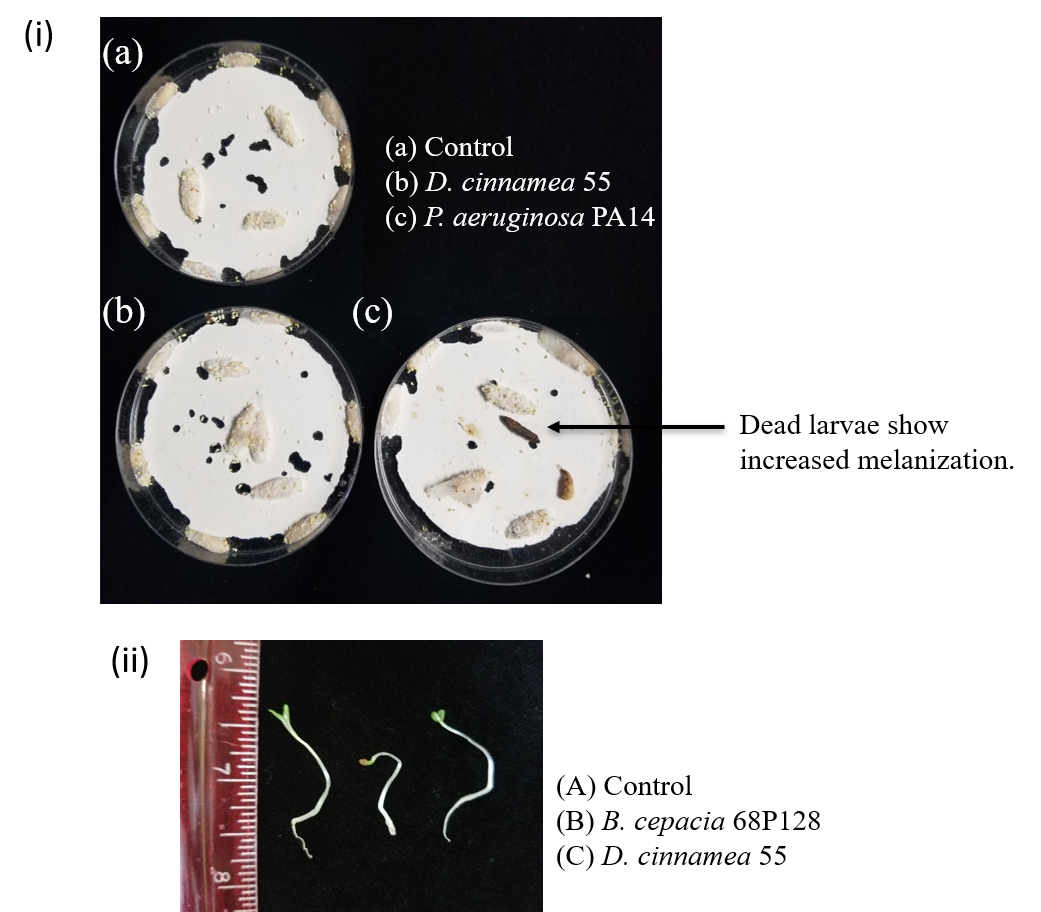

Supplement: FIGURE S3 — Photographs of Galleria mellonella assay plates (i), and alfalfa plants (ii) treated with D. cinnamea 55. [file Image_3.tif]
